# Selegiline, a monoamine oxidase-B inhibitor as a modulator of metabolic reprogramming for cancer therapy: a review

**DOI:** 10.3389/fphar.2026.1763962

**Published:** 2026-04-07

**Authors:** Jayanth Jayachandran, Sibin Nambidi, Suresh Babu Kondaveeti, Surajit Pathak, Arunkumar Radhakrishnan, Antara Banerjee, Asim K. Dutta Roy

**Affiliations:** 1 Department of Pharmacology, Chettinad Hospital and Research Institute (CHRI), Chettinad Academy of Research and Education (CARE), Chennai, India; 2 Medical Biotechnology lab, Faculty of Allied Health Sciences, Chettinad Academy of Research and Education (CARE), Chettinad Hospital and Research Institute (CHRI), Chennai, India; 3 Department of Biochemistry, Symbiosis Medical College for Women, Symbiosis International (Deemed University), Pune, India; 4 Department of Nutrition, Institute of Medical Sciences, Faculty of Medicine, University of Oslo, Oslo, Norway

**Keywords:** MAO-B inhibitor, metabolic reprogramming, mitochondrial dysfunction, selegiline, tumor microenvironment

## Abstract

Metabolic reprogramming plays a crucial role in cancer progression, therapeutic resistance, and tumor-microenvironment remodelling. Monoamine oxidase-B (MAO-B), a mitochondrial enzyme involved in oxidative deamination, has recently been identified as a metabolic regulator that influences reactive oxygen species (ROS) production, mitochondrial homeostasis, and redox-dependent signaling in tumors. Selegiline, an MAO-B inhibitor traditionally used in neurological disorders, is now gaining attention for its potential role in modulating tumor metabolism. Elevated MAO-B activity contributes to oxidative stress, genomic instability, immune suppression, and metabolic adaptations that support tumor survival. By inhibiting MAO-B, selegiline reduces ROS generation, alters mitochondrial respiration, regulates glycolytic flux, and disrupts hypoxia-associated pathways, making it a promising modulator of metabolic checkpoints in oncology. Relevant literature was collected from PubMed, Google Scholar, and ScienceDirect using keywords such as Selegiline, MAO-B inhibitor, tumor metabolism, oxidative stress, and drug repurposing in cancer. Relevant studies from the past 5 years, with inclusion criteria focusing on mechanistic, preclinical, and translational evidence related to MAO-B and selegiline-mediated metabolic regulation. Recent findings indicate that selegiline not only modulates cancer cell metabolism but also influences the tumor microenvironment by reducing inflammatory cytokine production, altering macrophage polarization, and enhancing susceptibility to therapeutic stress. Additionally, combination approaches with chemotherapeutics, metabolic inhibitors, and immunotherapies show synergistic potential. This review summarizes current insights into selegiline’s role in metabolic reprogramming, highlights existing challenges, and discusses future opportunities for repositioning selegiline as a targeted metabolic modulator in cancer therapy.

## 1 Introduction

Selegiline, an inhibitor of monoamine oxidase-B (MAO-B), was initially developed for the treatment of neurodegenerative diseases such as Parkinson’s disease and major depressive disorder due to its ability to prevent dopamine degradation in the central nervous system (Wang et al., 2023). In recent years, beyond its neurological applications, selegiline has gained attention for its favorable safety profile, well-characterized pharmacokinetics, and potential to modulate oxidative stress and mitochondrial metabolism (Rinaldi et al., 2023). Monoamine oxidases (MAOs) are mitochondrial flavoproteins that catalyse the oxidative deamination of biogenic amines, generating hydrogen peroxide as a by-product (Ostadkarampour and Putnins, 2021), thereby directly linking MAO activity to cellular redox regulation and energy metabolism, processes central to tumor metabolic reprogramming. Although MAO-B is predominantly expressed in neural tissues, its presence in peripheral organs and various tumors indicates a broader physiological role (Alsaad et al., 2024). Emerging evidence suggests that aberrant MAO-B expression promotes cancer progression by generating excessive reactive oxygen species (ROS), disrupting mitochondrial homeostasis, and modulating apoptotic and metabolic signaling networks.

Several preclinical studies have demonstrated that selegiline can influence multiple oncogenic pathways, including PI3K/Akt and NF-κB, while restoring redox balance and enhancing apoptotic sensitivity in tumor cells (Naoi et al., 2022; Hâncu et al., 2024). Beyond its metabolic role, MAO-B has also emerged as an important regulator within the tumor microenvironment (TME) (Chen et al., 2023). By targeting the mitochondrial enzyme that directly influences cellular redox metabolism, selegiline potentially acts as an emerging metabolic checkpoint inhibitor, altering cancer cell adaptation to oxidative stress and nutrient competition within the TME. Although based on current preclinical evidence, selegiline should be regarded as an emerging metabolic checkpoint modulator, as its clinical validation in this context remains to be established. Elevated MAO-B expression has been reported in glioblastoma, prostate, breast, and hepatocellular carcinomas, supporting its role as a metabolic driver in diverse cancers (Aljanabi et al., 2021). These findings open new avenues for repositioning MAO-B inhibitors, such as selegiline supplementation to mainstream therapy, potentially enhancing their efficacy by modulating oxidative stress and apoptotic thresholds in tumor cells.

Growing attention has been directed toward the broader class of MAO inhibitors in cancer biology. Although MAO-A and MAO-B were traditionally studied in the context of neurotransmitter metabolism, accumulating evidence indicates that both isoforms contribute to tumorigenesis through distinct redox-driven and metabolic mechanisms, supporting the rationale for repurposing approved neurological drugs for oncology (Siddiqui et al., 2022). Comparative studies indicate that selective MAO-B inhibitors such as selegiline, reversible inhibitors like safinamide, and non-selective MAO inhibitors, including phenelzine and tranylcypromine, differ substantially in their biochemical specificity, mitochondrial effects, and influence on metabolic rewiring within the tumor microenvironment, demonstrating a growing interest in exploring MAO inhibitors in cancer medicine (Sblano et al., 2024). Understanding these differences is essential for positioning selegiline within the evolving therapeutic landscape and for evaluating how MAO inhibition shapes cancer cell behavior, redox homeostasis, and treatment responsiveness.

Recent advances in nanotechnology and drug delivery systems have opened up new opportunities to enhance selegiline’s tumor-specific accumulation, bioavailability, and overall therapeutic efficacy. Such nanoformulations may potentiate metabolic modulation within the TME, thereby promoting synergistic effects when combined with chemotherapeutic or immunomodulatory agents (Wang et al., 2025; Eslami et al., 2024). Thus, the repositioning of selegiline is not only a cost-effective approach but also a scientifically convincing strategy that aligns with precision oncology and the growing emphasis on metabolic reprogramming in cancer treatment (Satish et al., 2023). In this review, we propose an integrated framework in which MAO-B-dependent and MAO-independent actions of selegiline integrate to modulate tumor metabolism and immune reprogramming. MAO-B-derived hydrogen peroxide and aldehydes drive redox imbalance, stabilize HIF-1α, and activate NF-κB, thereby promoting glycolytic shifts, mitochondrial adaptations, angiogenesis, and an immunosuppressive tumor microenvironment. In parallel, MAO-independent activities of selegiline, including direct disruption of mitochondrial respiration and modulation of signaling pathways such as PKC, can aggravate bioenergetic stress and apoptosis, particularly in metabolically vulnerable tumors.

Unlike previous MAO or MAO-B reviews, we specifically examined recent evidence that positions selegiline as a metabolic checkpoint modulator and integrated this with pharmacokinetics, metabolic phenotypes, and biomarker-guided patient selection. We also highlight emerging data on MAO-B expression heterogeneity across tumor types, MAO-independent mitochondrial actions of selegiline, and rational combination or nanodelivery strategies, which have not been reviewed collectively before. So, this review aims to consolidate current evidence on the emerging role of selegiline, a selective MAO-B inhibitor, in cancer therapy, with particular emphasis on MAO-B-dependent and MAO-independent mechanisms that shape metabolic reprogramming, oxidative stress responses, and tumor-immune interactions.

## 2 Role of monoamine oxidases in tumor metabolism and tumor microenvironment modulation

**FIGURE 1:**
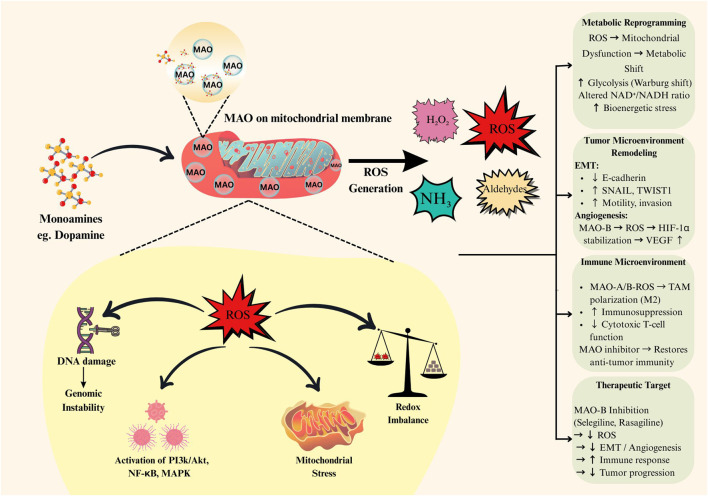
Role of MAO-Derived ROS in promoting Oncogenic Signalling pathways and Tumor Progression. Overview of the oncogenic role of MAO-B in cancer cells. Elevated MAO-B activity increases mitochondrial oxidative deamination of monoamines, leading to excessive generation of hydrogen peroxide and ROS. The resulting redox imbalance promotes DNA damage, mitochondrial dysfunction, metabolic remodelling, and activation of pro-tumorigenic signaling pathways, including PI3K/Akt and NF-κB. These events collectively support tumor growth, survival, migration, and resistance to therapy.

MAO-B is a mitochondrial outer membrane enzyme primarily involved in the oxidative deamination of monoamines such as dopamine, producing hydrogen peroxide (H_2_O_2_), ammonia, and corresponding aldehydes (Wang et al., 2012). Beyond ROS generation, the monoamine substrates metabolized by MAO-B themselves may influence tumor progression. Neurotransmitters such as dopamine and trace amines like phenethylamine can modulate cellular signaling pathways through receptor-mediated mechanisms that regulate proliferation, angiogenesis, and immune responses. Alterations in monoamines may indirectly contribute to metabolic reprogramming by influencing intracellular signaling networks, mitochondrial activity, and redox homeostasis within tumor cells and the tumor microenvironment (Ostadkarampour and Putnins, 2021; Hâncu et al., 2024; Aljanabi et al., 2021). While MAO-Bs have been extensively studied in the context of neurodegenerative diseases, accumulating evidence indicates their significant involvement in tumorigenesis (Shih, 2018). The enzymatic activity of MAO-B leads to increased ROS levels, particularly H_2_O_2_, which contribute to oxidative stress and DNA damage, ultimately fostering genetic mutations and genomic instability, the key hallmarks of cancer initiation (Shar et al., 2015). Furthermore, MAO-B-derived ROS activate oncogenic signaling pathways, including PI3K/Akt, NF-κB, and MAPK, thereby promoting cell proliferation, angiogenesis, and resistance to apoptosis (Nakamura and Takada, 2021). Beyond intracellular effects, MAO-B activity also alters the TME by modulating redox balance and inflammatory responses (Wang Y.-C. et al., 2021). The biochemical activity of MAO-B at the mitochondrial outer membrane and its downstream effects on redox signaling, metabolic reprogramming, and tumor microenvironment modulation are schematically illustrated in Figure 1. MAO-B-driven oxidative stress contributes to extracellular matrix remodeling, stromal activation, and enhanced metastatic potential. In addition, prolonged MAO-B-mediated oxidative stress induces mitochondrial damage and triggers compensatory metabolic adaptations, including increased glycolysis and altered NAD^+^/NADH balance (Chen L. et al., 2020).

MAO-B is highly expressed in several cancer types, including human triple-negative breast cancer (TNBC), lung cancer, glioblastoma, colorectal cancer (CRC), non-small cell lung cancer (NSCLC), and hepatocellular carcinoma (Esmaeili et al., 2021). In CRC, one of the most prevalent malignancies globally, reported that MAO-B expression was significantly higher in tumor tissues compared to adjacent normal tissues, and that its upregulation correlated with tumor recurrence, poor prognosis, and increased expression of epithelial-to-mesenchymal transition (EMT)-related genes (Hossain et al., 2022). These findings highlight the clinical relevance of MAO-B overexpression across multiple tumor types. A comparative summary of MAO-B expression patterns, functional significance, and therapeutic responsiveness across cancers is provided in Table 1.

**TABLE 1:** Expression and functional impact of MAO-B in different cancers: prognostic and therapeutic implications, with dominant mechanistic category (redox, metabolism, immune/TME) for each tumor type.

Cancer type	MAO-B expression	Functional/Clinical impact	Prognostic relevance	Sensitivity to MAO-B inhibition	Primary mechanistic category (redox/metabolism/immunomodulation)	References
Prostate	Upregulated	Facilitates tumor growth, hormone independence, and stromal metabolic support	Associated with progression and drug resistance	Sensitization observed in preclinical models	Redox + metabolism; stromal/TME	Chen et al. (2023)
Colorectal cancer	High	Enhances metastasis, drives EMT, and redox imbalance	Associated with aggressive disease	Early evidence supports combinatorial use	Redox + metabolism (EMT)	Yang et al. (2020)
Non-small cell lung cancer	Upregulated	Mediates cellular radioresistance via NF-κB and hydrogen peroxide	Predicts lower response to therapy	Improves radiosensitivity in models	Redox (ROS–NF-κB)	Son et al. (2016)
Breast cancer (TNBC)	High	Promotes EMT, invasion, and metabolic plasticity	Predicts risk of recurrence, particularly in TNBC	Targetable in the triple-negative subtype	Redox + metabolism	Sun et al. (2017)
Glioblastoma	High	Drives ROS production, promotes survival under hypoxia	Linked to poor clinical outcomes, high HIF-1α	High promising preclinical results	Redox + hypoxia/metabolism	Shar et al. (2015)
Oral SCC	Low	Minimal metabolic involvement	Limited prognostic significance	Not established	Limited MAO-B involvement	Sharif Siam et al. (2020)
Gastric cancer	Low	Indirect effect, heightened NE suggests alternative metabolic targets	Unclear due to low MAO-B, high adrenergic signaling	Focus on adrenergic modulation	Adrenergic/non-MAO-B metabolism	Wang et al. (2021a)

Under normoxic or hypoxic conditions, MAO-derived ROS stabilize Hypoxia-inducible factor 1-alpha (HIF-1α) and stimulate pro-growth signaling pathways such as AKT and extracellular signal-regulated kinase (ERK), promoting proliferation and metabolic shifts that support biomass accumulation. In various cell models, MAO inhibition has been shown to decrease proliferation (Resta et al., 2022). In prostate and other tumor types, MAO activity promotes the transcription of EMT regulators, including TWIST1 and SNAIL, leading to loss of epithelial markers and increased cellular motility. These effects are mediated through ROS-dependent kinase activation and redox-sensitive transcriptional networks governing EMT (Nordio et al., 2023).

Collectively, these observations establish MAO-B as more than a housekeeping metabolic enzyme and suggest its broader role in shaping tumor biology through redox-linked mechanisms. However, MAO-B expression levels and isoform balance with MAO-A vary substantially across tumor types such as TNBC, CRC, NSCLC, glioblastoma, oral squamous cell carcinoma (OSCC), and hepatocellular carcinoma, indicating that MAO-B-driven effects may be context-dependent rather than universal. Despite growing evidence, most data are derived from preclinical models, and direct comparative studies across tumors with high versus low MAO-B expression remain limited. It therefore remains unclear whether MAO-B acts as a primary oncogenic driver or as a context-dependent amplifier of existing metabolic and microenvironmental alterations. This distinction forms the conceptual basis for the subsequent sections that examine MAO-B-dependent and MAO-independent mechanisms relevant to therapeutic targeting. In contrast to earlier descriptive overviews of MAO biology, this section emphasizes MAO-B as a context-dependent metabolic and microenvironmental regulator and explicitly outlines testable hypotheses about when MAO-B acts as a driver versus an amplifier of oncogenic metabolism and immune escape.

## 3 MAO-B-derived reactive oxygen species as a central integrator of tumor metabolism and the tumor microenvironment

ROS produced by MAO-B are implicated in redox signaling, which originates from the outer mitochondrial membrane, and are responsible for the integration of tumor metabolism with the TME. MAO-B-derived ROS are implicated in metabolic adaptation, survival signaling, and TME remodeling by inducing reactive stroma, extracellular matrix changes, and pro-tumorigenic phenotypes, as shown in prostate cancer models, where stromal MAO-B enhances tumor growth by ROS-mediated pathways (Pu et al., 2024). In bladder cancer cells, MAO-B is implicated in glucose-dependent energy metabolism and proliferation by ROS production, linking metabolic reprogramming with tumor progression (Resta et al., 2022). The overexpression of MAO-B in various cancers is linked to enhanced ROS production, which supports oncogenic metabolism and immune modulation in the TME, making MAO-B a potential therapeutic target (Alsaad et al., 2024). ROS in the TME have a biphasic effect: moderate concentrations enhance tumor progression and immune modulation, whereas high concentrations of ROS trigger cancer cell death; this phenomenon is vital for tumor development and responsiveness to therapy (Iqra et al., 2025). MAO-B-derived ROS are a promising target for inhibiting tumor metabolic signaling and TME remodelling to suppress cancer growth.

### 3.1 ROS-driven metabolic reprogramming and stress adaptation in tumor cells

MAO-B-derived ROS play a role in metabolic reprogramming and stress responses in tumor cells by activating redox-sensitive pathways, leading to genomic instability and metabolic plasticity. The continuous production of H_2_O_2_ stabilizes hypoxia-inducible factor-1α (HIF-1α) under both hypoxic and normoxic conditions, thereby favoring glycolysis and increasing glucose uptake, as seen in bladder cancer cells, where MAO inhibition attenuates glycolysis and proliferation (Resta et al., 2022). The activation of kinase pathways, such as PI3K/Akt, MAPK, and NF-κB, contributes to tumor cell proliferation, resistance to apoptosis, and adaptation to metabolic stress. These ROS-mediated signals also trigger epithelial-to-mesenchymal transition (EMT) through the transcription factors SNAIL and TWIST, increasing the invasiveness, metastatic potential, and resistance to therapy in various cancers (Wang et al., 2021b). The overexpression of MAO-B and the production of ROS are associated with poor prognosis, recurrence, and resistance to chemotherapy or radiotherapy in colorectal cancer, non-small cell lung cancer, glioma, and triple-negative breast cancer (Rapozzi et al., 2025). In summary, MAO-B-derived ROS are key mediators of tumor metabolic reprogramming and stress adaptation by converging redox signals with oncogenic pathways (Li et al., 2025).

### 3.2 ROS-mediated remodeling of the tumor microenvironment

MAO-B-related ROS contribute substantially to the remodeling of the TME by influencing cytokine and chemokine expression via redox-regulated transcriptional networks. The sustained ROS signaling pathway is also involved in angiogenesis induction via HIF-1α-mediated VEGF transcription, leading to dysregulated vascular remodeling and, in turn, favoring tumor growth (Cheung and Vousden, 2022; Shen et al., 2025). The high oxidative stress in the TME inhibits cytotoxic T-cell and natural killer cell function while promoting the differentiation of tumor-associated macrophages into an immunosuppressive M2-like phenotype, thereby mediating immune evasion (Kuo et al., 2022).

Cancer-associated fibroblasts (CAFs) also display enhanced extracellular matrix production and metabolic support for tumor cells due to ROS, thereby contributing to resistance to successful antitumor immunity (Kennel and Greten, 2021). These ROS-induced changes cumulatively create a tumor-supportive microenvironment by synergistically combining the metabolic, immune, and stromal aspects of the TME. The modulation of ROS dynamics in the TME has shown promise for improving cancer therapy by regulating immune responses and disrupting tumor-stromal interactions (Weinberg et al., 2019).

### 3.3 ROS as a convergent mechanistic axis linking metabolism and immunomodulation

**FIGURE 2:**
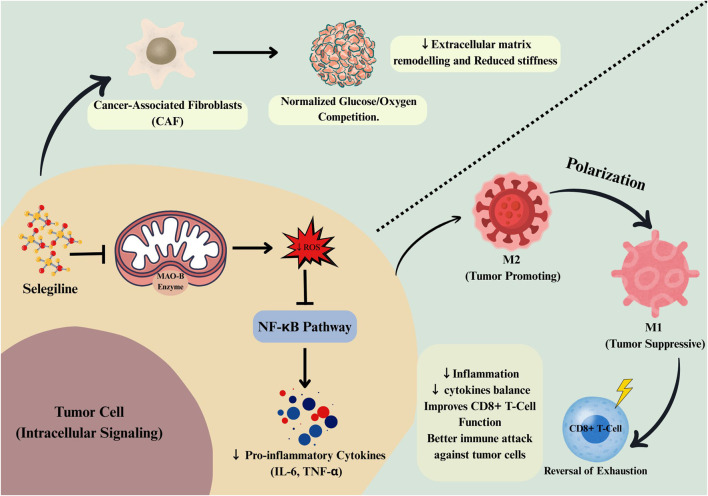
MAO-B-derived reactive oxygen species as a central hub linking tumor, immune, and stromal metabolism. MAO-B activity at the outer mitochondrial membrane generates reactive oxygen species (ROS) that activate redox-sensitive signaling pathways such as NF-κB, promoting pro-inflammatory cytokine production and metabolic adaptation within tumor cells. In the tumor microenvironment, MAO-B–associated oxidative stress supports cancer-associated fibroblast (CAF) activation, extracellular matrix remodeling, and metabolic competition for glucose and oxygen. Elevated ROS also promote immunosuppressive macrophage polarization (M2) and T-cell exhaustion. Inhibition of MAO-B by selegiline reduces ROS signaling, normalizes stromal metabolism, shifts macrophages toward a pro-inflammatory M1 phenotype, and restores cytotoxic T-cell function, collectively remodeling the tumor microenvironment toward an antitumor state.

The ROS produced by MAO-B plays a crucial role as a convergent signaling platform that integrates tumor metabolism, hypoxia response, angiogenesis, immune evasion, and stroma remodeling. This phenomenon underlies the convergence of metabolic reprogramming and immune modulation in MAO-B-driven cancers, in which ROS stimulate glycolysis, stabilize HIF-1α, induce angiogenic factors such as VEGF, and regulate immune cell function and stroma activation (Pu et al., 2024). In the prostate cancer stroma, MAO-B increases ROS production, which stimulates extracellular matrix remodeling and pro-tumorigenic signaling via the CXCL12/CXCR4 axis, promoting tumor development. Blocking MAO-B, including through pharmacological inhibition with agents such as selegiline, or disrupting ROS-mediated signaling, suppresses tumor metabolic fitness and reprograms the TME, inhibiting proliferation and immune evasion (Resta et al., 2022). These observations provide a rationale for the therapeutic use of MAO inhibitors, such as selegiline, to target metabolic and immune vulnerabilities in various cancers (Cheung and Vousden, 2022). The downstream consequences of MAO-B-derived ROS are essential for understanding the role of mitochondrial and signaling pathway contributions to cancer vulnerabilities in various tumor microenvironments (Alsaad et al., 2024). These integrated tumor-intrinsic and microenvironmental effects of MAO-B-derived ROS are schematically summarized in Figure 2.

## 4 Relative effectiveness of MAO inhibitors in cancer therapy

MAOIs have emerged as a promising area of increasing interest in oncology due to their ability to modulate monoamine metabolism and mitochondrial redox processes. The therapeutic impact of MAOIs depends primarily on isoform selectivity (MAO-A vs. MAO-B) and the mode of enzyme inhibition (reversible vs. irreversible), which together influence duration of action, systemic exposure, and safety profiles (Li M. et al., 2023). Selective MAO-B inhibitors, such as selegiline and rasagiline, irreversibly inhibit MAO-B, resulting in sustained suppression of enzyme activity, whereas reversible inhibitors, such as safinamide, allow transient modulation of MAO-B function (Kundu et al., 2024; Luo et al., 2023; Ge et al., 2025). These pharmacological differences are particularly relevant in cancer, where prolonged versus tunable enzyme inhibition may differentially affect tumor metabolism and treatment scheduling.

Non-selective inhibitors, including phenelzine and tranylcypromine, inhibit both MAO-A and MAO-B isoforms, thereby modulating intracellular redox homeostasis, catecholamine turnover, and apoptotic signaling (Ma et al., 2024). Also, this dual inhibition enhances antiproliferative effects across several cancer models, but it is associated with systemic side effects that limit its long-term clinical application (McLeod et al., 2020). Furthermore, phenelzine has demonstrated effects in a phase II clinical evaluation in hormone-resistant prostate cancer, providing proof-of-concept that MAO targeting can yield clinical benefit in cancer (Wang et al., 2020). Additionally, tranylcypromine is a characteristic model of a repurposed dual-function MAOI, acting as both an MAO inhibitor and an inhibitor of lysine-specific demethylase 1 (LSD1) (Matveychuk et al., 2021). Key biochemical and pharmacological distinctions among major MAO inhibitors are summarized in Table 2.

**TABLE 2:** Comparison of MAO inhibitors used in cancer therapy, including isoform selectivity, reversibility, cancer models, mechanistic targets, primary mechanistic category (redox, metabolism, immune/TME), and clinical/preclinical status.

Inhibitor	Selectivity	Reversibility	Cancer models studied	Primary mechanistic category (redox/metabolism/immune)	Clinical/Preclinical status	References
Selegiline	MAO-B selective	Irreversible	Prostate, breast, glioma, AML	Redox + metabolism (mitochondrial)	Preclinical, repurposing in progress	Matveychuk et al. (2021), Liu et al. (2025)
Rasagiline	MAO-B selective	Irreversible	Glioblastoma	Redox/HIF-1α signaling	Preclinical	Naoi et al. (2022)
Phenelzine	Non-selective (A/B)	Irreversible	Prostate cancer	Redox + metabolism; indirect immune	Phase II clinical trial	Park et al. (2019); Wu et al., (2023)
Tranylcypromine	Non-selective (A/B) + LSD1 inhibitor	Irreversible	Gastric, leukemia	Epigenetic + metabolism	Preclinical	Skorzynski et al. (2025), Magyar and Szende (2004)
Safinamide	MAO-B selective	Reversible	Limited cancer data	Redox modulation	Preclinical	Jiang et al. (2019), Borštnar et al. (2011)

Comparative studies demonstrate that selective MAO-B inhibitors generally offer superior pharmacological selectivity and safety margins, particularly in tumors characterized by elevated MAO-B expression. In contrast, non-selective MAOIs may induce broader cytotoxic effects but are more frequently associated with systemic adverse events, limiting their long-term clinical utility (Park et al., 2019; Liu et al., 2025). However, irreversible inhibitors such as selegiline produce sustained enzyme inactivation, whereas reversible inhibitors provide greater temporal control, which may be advantageous in combination regimens (Liang et al., 2025). Importantly, the effects of MAOIs extend beyond tumor cells to the surrounding microenvironment. When MAO activity is inhibited (for example, by MAO-B inhibitors), less hydrogen peroxide is produced, and oxidative stress decreases; downstream pathways, such as HIF-1α signaling, are modulated (Wu et al., 2023). This redox regulation has implications for metabolic competition between cancer and immune cells, as excessive ROS can impair immune cell metabolism and function (Skorzynski et al., 2025). Overall, comparative analyses of MAO inhibitors highlight a critical balance between efficacy, selectivity, and tolerability. Collectively, this section emphasizes mechanistic distinctions-such as mitochondrial targeting, dual MAO/LSD1 inhibition, and immunometabolic effects-that inform the rational prioritization of selegiline and other MAO inhibitors across specific cancer and metabolic phenotypes.

## 5 Pharmacokinetics and mechanistic basis of selegiline relevant to tumor metabolism

### 5.1 Pharmacokinetic properties and dose-dependent MAO selectivity

Selegiline acts as a mechanism-based, irreversible inhibitor of MAO-B at therapeutic concentrations. Also, higher systemic exposures can result in partial inhibition of MAO-A in addition to MAO-B (Magyar and Szende, 2004). This dose-dependent selectivity underlies its established neuroprotective activity and has important implications for its potential anticancer effects (Jiang et al., 2019). Selegiline irreversibly inhibits MAO-B by covalently binding to the flavin adenine dinucleotide (FAD) cofactor within the enzyme’s active site, thereby preventing oxidative deamination of monoamines (Borštnar et al., 2011). Through this mechanism, selegiline may reduce oxidative stress-induced DNA damage, metabolic reprogramming, and oncogenic signaling pathways stimulated by MAO overactivity in tumor cells by lowering intracellular ROS levels (Szökő et al., 2018).

Following oral administration, selegiline is rapidly absorbed, achieving peak plasma concentrations within 0.5–2 h (Chew et al., 2021). Its lipophilic nature facilitates penetration across the blood-brain barrier and distribution to peripheral tissues, including platelets, hepatocytes, and tumor cells, where MAO-B is also expressed (Gealageas et al., 2018). Although selegiline has a relatively short plasma half-life (approximately 1.5–3 h), irreversible MAO-B inhibition leads to prolonged functional suppression of enzyme activity, with recovery dependent on *de novo* enzyme synthesis over 1–2 weeks (Liang et al., 2025).

At lower doses (5–10 mg/day), selegiline selectively inhibits MAO-B, whereas partial MAO-A inhibition occurs at higher systemic concentrations (>20 mg/day) (Mahmood, 1997). In tumor tissues, such dual inhibition may influence therapeutic outcomes in tumors expressing both MAO isoforms, but it may also increase the risk of off-target effects (Fowler et al., 2015). Consequently, careful dose selection and scheduling are essential, particularly in combination regimens. Potential drug-drug interactions should also be considered, as selegiline undergoes hepatic metabolism involving cytochrome P450 enzymes that may overlap with those used by certain chemotherapeutic agents (Guglielmi et al., 2022; Salonen et al., 2003).

### 5.2 Mitochondrial localization of MAO-B and implications for tumor metabolism

MAO-B is localized to the outer mitochondrial membrane, positioning selegiline to influence mitochondrial redox balance and cellular energy homeostasis directly (Alsaad et al., 2024). By inhibiting MAO-B at this strategic site, selegiline alters mitochondrial-associated metabolic signaling and apoptosis-related pathways that are highly relevant to cancer cell survival (Sorato et al., 2014). Rather than providing general pharmacology, this section emphasizes pharmacokinetic and mitochondrial features of selegiline that are most relevant for oncology, including dose-dependent MAO-A/B selectivity, tissue distribution beyond the CNS, and opportunities to exploit its prolonged enzyme inactivation and Nano formulations for tumor-selective metabolic targeting. This mitochondrial localization provides a mechanistic rationale for the observed effects of selegiline on tumor bioenergetics and apoptotic sensitivity, which are further elaborated in subsequent sections. Importantly, these mitochondrial modulatory effects underpin both MAO-B-dependent and MAO-B-independent actions of selegiline, linking its pharmacokinetic properties to its metabolic and anticancer potential (Somnath Kumkar et al., 2024).

## 6 Selegiline-induced metabolic reprogramming in cancer

### 6.1 MAO-B-dependent mechanisms: redox-linked metabolic and stress adaptation

Selegiline exerts substantial anticancer activity by inhibiting MAO-B-dependent redox signaling. As detailed in Section 4, MAO-B activity generates hydrogen peroxide that functions as a signaling mediator linking monoamine metabolism to tumor metabolic reprogramming and stress adaptation. In tumors with elevated MAO-B expression, this redox axis promotes aggressive phenotypes, therapeutic resistance, and metabolic plasticity (Alsaad et al., 2024).

Increased expression of MAO-B has been shown in various cancers, associated with aggressive clinical behaviour and drug resistance. In CRC, MAO-B was significantly overexpressed in tumor tissues compared with adjacent normal tissues. This overexpression was associated with poor prognosis, recurrence, and increased expression of EMT-related genes (Yang et al., 2020). Additionally, NSCLC, MAO-B mRNA, and protein levels were found to increase in a dose-dependent manner following ionizing radiation, contributing to radioresistance by activating stress-responsive survival pathways (Zhang et al., 2020; Jayachandran et al., 2025). Pharmacological inhibitors of MAO-B suppress these pathways and enhance radiosensitivity, an effect further supported by studies using MAO-B-targeting compounds such as danshensu (Son et al., 2016). These observations are consistent with recent studies linking MAO-B expression to metabolic plasticity and therapeutic vulnerability, particularly in gliomas, where mitochondrial-targeted strategies are being explored to exploit OXPHOS dependence and redox stress (Gatto et al., 2024). In CRC and NSCLC, MAO-B-derived H_2_O_2_ stabilizes HIF-1α and activates NF-κB, which, in turn, upregulates EMT-related transcription factors and survival genes, linking MAO-B activity to invasion, recurrence, and radioresistance (Yang et al., 2020; Zhang et al., 2020).

Elevated MAO-B expression has also been reported in gliomas, where it correlates with hypoxia-associated signaling and mitochondrial metabolic dependence, reinforcing the link between MAO-B activity and redox-driven tumor adaptation (Shar et al., 2015; Ryu et al., 2018; Marconi et al., 2019). Collectively, these findings indicate that MAO-B-dependent mechanisms dominate in tumor contexts characterized by high MAO-B expression and oxidative stress, including colorectal cancer, non-small cell lung carcinoma, triple negative breast cancer, and glioma.

### 6.2 MAO-B-independent mechanisms: Mitochondrial and signaling disruption

Beyond redox-mediated effects, selegiline also exhibits MAO-B-independent anticancer activity. In breast cancer models (MCF-7 and MDA-MB-231), selegiline induces apoptosis by inhibiting protein kinase C (PKC) phosphorylation, independent of oxidative stress or MAO-B activity. Thus, PKC inhibition and downstream apoptosis represent MAO-B-independent signaling actions of selegiline that operate even when MAO-B activity is not the primary driver (Somnath Kumkar et al., 2024). This identifies a non-canonical signaling mechanism through which selegiline can trigger tumor cell death even when MAO-B is not dominant.

Furthermore, in acute myeloid leukemia (AML) cells, selegiline was shown to impair mitochondrial respiration, reduce ATP levels, and downregulate genes involved in mitochondrial function and glycolysis. Importantly, these effects were observed even in the absence of MAO-B inhibition, suggesting that selegiline may act as a direct mitochondrial disruptor. These observations point to an MAO-B-independent, mitochondria-targeted effect of selegiline. In AML cells, loss of mitochondrial respiration and ATP depletion following selegiline exposure suggest disruption of electron transport and bioenergetic sensing pathways, consistent with a direct mitochondrial toxin-like effect (Ryu et al., 2018). Such MAO-B-independent mitochondrial effects may be particularly relevant in malignancies with low or variable MAO-B expression, including AML and selected breast cancer subtypes.

### 6.3 Tumor-specific MAO-B expression and context dependence

Furthermore, tumor-type-specific expression patterns further modulate selegiline responsiveness. In breast cancer, MAO-B expression is significantly elevated in TNBC compared with luminal subtypes, where MAO-A predominates, suggesting a selective therapeutic window for MAO-B inhibition in TNBC (Sun et al., 2017). However, OSCC and gastric cancer exhibited decreased MAO-B expression, indicating limited relevance of MAO-B-dependent mechanisms and a potential preference for alternative metabolic or adrenergic targets in these tumors (Sharif Siam et al., 2020; Wang et al., 2021a; Oh et al., 2020).

**FIGURE 3:**
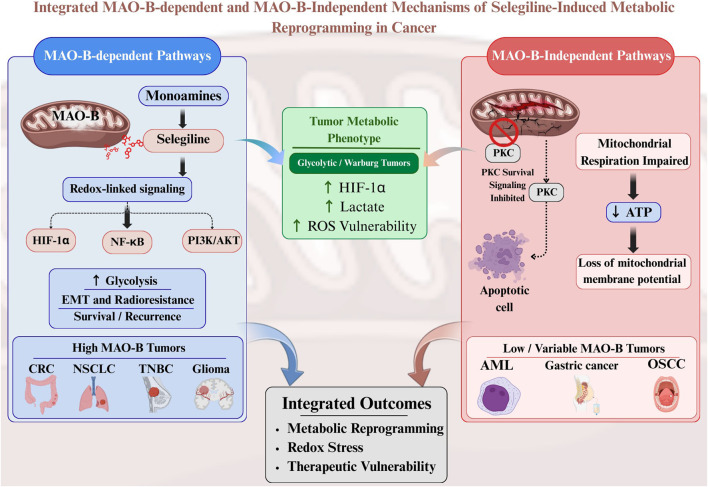
Integrated MAO-B-dependent and MAO-B-independent mechanisms of selegiline-induced metabolic reprogramming in cancer. Schematic representation of MAO-B-dependent and MAO-B-independent mechanisms underlying selegiline-induced metabolic reprogramming in cancer. In MAO-B-high tumors, selegiline modulates monoamine-driven redox signaling, influencing HIF-1α and PI3K/Akt pathways to promote glycolytic dependence and ROS vulnerability. In contrast, in tumors with low or variable MAO-B expression, selegiline primarily induces mitochondrial dysfunction, ATP depletion, and apoptosis independent of MAO-B inhibition. These context-dependent mechanisms converge to enhance redox stress and therapeutic vulnerability across tumor types.

Thus, MAO-B-dependent ROS/HIF-1α signalling is most likely to dominate in high-MAO-B expressing tumors such as TNBC, CRC, NSCLC, and glioma, whereas PKC and mitochondrial mechanisms may be more relevant in AML, breast models with low MAO-B, OSCC, and gastric cancer (Shar et al., 2015; Sun et al., 2017; Sharif Siam et al., 2020; Wang et al., 2021a; Ryu et al., 2018; Marconi et al., 2019; Oh et al., 2020). Overall, these findings show that selegiline acts as a putative metabolic checkpoint inhibitor that can reprogram tumor metabolism by regulating redox balance, disrupting mitochondrial function, and controlling hypoxia pathways. These expression patterns support a model in which MAO-B-dependent redox signaling predominates in MAO-B-high tumors, whereas MAO-B-independent mitochondrial and signaling effects of selegiline may contribute more substantially in tumor contexts characterized by low MAO-B expression or activity. These tumor-specific expression patterns support a context-dependent model in which MAO-B-dependent redox signaling and MAO-B-independent mitochondrial mechanisms differentially contribute to selegiline responsiveness, as summarized schematically in Figure 3.

### 6.4 Metabolic phenotype-specific responses to selegiline

Cancer cells exhibit heterogeneous metabolic phenotypes ranging from glycolysis-dominant “Warburg” states to oxidative phosphorylation (OXPHOS)-dependent profiles. These metabolic phenotypes may influence whether MAO-B-dependent or MAO-B-independent effects predominate (Pa et al., 2025; Park et al., 2020; Chelakkot et al., 2023; Liao et al., 2023). In glycolytic, HIF-1α-driven tumors, disruption of MAO-B-associated redox signaling destabilizes metabolic support for rapid proliferation and stress tolerance (Du et al., 2020). In contrast, OXPHOS-dependent tumors rely on intact mitochondrial respiration, rendering them particularly sensitive to selegiline-mediated mitochondrial dysfunction and ATP depletion (Frattaruolo et al., 2020; Mahmoud and Karim, 2025; Du et al., 2025). Linking these metabolic phenotypes to MAO B expression provides a mechanistic basis for predicting whether ROS/HIF-1α-driven or mitochondrial mechanisms of selegiline will predominate in a given tumor.

Incorporating metabolic phenotyping, including assessments of glycolytic capacity, OXPHOS dependence, and hypoxia signaling, will be essential for predicting tumor responsiveness and designing rational combination strategies. Linking metabolic phenotype with MAO-B expression provides a mechanistic framework to determine whether MAO-B-dependent or MAO-B-independent actions of selegiline are likely to predominate in a given tumor context.

### 6.5 Integrated mechanistic framework and limitations

Together, these findings indicate that selegiline modulates tumor progression through both MAO-B-dependent and MAO-independent mechanisms that converge on metabolic reprogramming and redox stress. MAO-B-dependent pathways, driven by H_2_O_2_ production and downstream HIF-1α/NF-κB activation, promote EMT, radioresistance, and hypoxia-linked metabolic shifts in tumors such as CRC, NSCLC, TNBC, and glioma, whereas MAO-independent actions, including PKC inhibition and direct mitochondrial respiratory disruption, become particularly relevant in settings like AML and breast cancer models with low or variable MAO-B expression.

In glycolytic, HIF-1α-driven “Warburg” tumors, MAO-B inhibition primarily destabilizes redox homeostasis and glycolytic support, whereas in OXPHOS-addicted cancers, selegiline’s mitochondrial effects precipitate energetic collapse and apoptosis. This integrated framework suggests that the relative contribution of MAO- B-dependent versus MAO-independent mechanisms is dictated by tumor-intrinsic metabolic phenotype and MAO B expression status, underscoring the need for biomarker-guided patient selection and rational combination strategies. Thus, MAO-B-dependent and MAO-independent actions of selegiline are not mutually exclusive but can operate in parallel, with their relative dominance determined by MAO-B expression, hypoxic signaling, and the glycolytic versus OXPHOS metabolic phenotype of individual tumors. These mechanisms may operate in parallel, with their relative contributions dictated by tumor-intrinsic MAO-B expression, metabolic phenotype, and microenvironmental context, as summarized in Table 3. However, most current evidence derives from *in vitro* and limited *in vivo* models, and the relative contribution of these mechanisms has not been systematically dissected using genetic loss-of-function approaches. Tumor-specific validation will therefore be essential before broadly generalizing selegiline’s metabolic actions across cancer types.

**TABLE 3:** Conceptual framework of MAO-B-dependent and MAO-independent actions of selegiline in cancer.

Mechanism of action	MAO-B-dependent metabolic effects	MAO-B-independent mitochondrial/signaling effects	Dominant tumor types involved	Tumor types	References
Redox/ROS	H_2_O_2_ from monoamine deamination; ROS-driven DNA damage; HIF-1α and NF-κB activation; EMT and angiogenesis	Direct mitochondrial ROS modulation altered antioxidant systems without MAO-B requirement	MAO-B-high, hypoxic tumors	Glioblastoma, CRC, NSCLC, TNBC	Alsaad et al. (2024), Shar et al. (2015), Nakamura and Takada (2021), Yang et al. (2020)
Metabolism	Glycolytic shift, altered NAD^+^/NADH, supports radio/chemoresistance	Inhibition of mitochondrial respiration, ↓ATP, downregulation of OXPHOS/glycolysis genes, and PKC inhibition	MAO-B-dependent in MAO-B-high; MAO-independent in MAO-B-low or AML/breast models	CRC, NSCLC, TNBC vs. AML, some breast cancers	Hâncu et al. (2024), Chen L. et al. (2020), Resta et al. (2022), Ryu et al. (2018)
Immune/TME	ROS-driven cytokine induction, TAM M2 polarization, and immunosuppressive TME	Selegiline-driven changes in immune cell metabolism, apoptosis, and signaling beyond MAO-B	MAO-B-dependent in MAO-B-high, inflamed TMEs; MAO-independent, where immune cells are directly targeted	HCC, breast cancer, prostate models	Ostadkarampour and Putnins (2021), Wang et al. (2021a), Pu et al. (2024), Ma et al. (2024)

## 7 Effects of selegiline in preclinical cancer models

Selegiline has been investigated in several preclinical oncology studies, highlighting its potential in anticancer activity. However, these studies are less extensive compared to their neurological research. Emerging evidence indicates that selegiline can influence cancer cell survival by targeting both MAO-dependent and MAO-independent mechanisms, as outlined in Section 5, although the overall preclinical evidence base remains less extensive than that for its neurological applications (Sblan et al., 2024; Wei and Wu, 2024). High-dose selegiline has been reported to induce cytotoxicity in various cancer cell lines, such as melanoma and acute myelogenous leukemia, primarily through mitochondrial dysfunction and inhibition of cellular respiration, leading to apoptosis (Ryu et al., 2018; Wei and Wu, 2024). However, these effects are generally observed at high concentrations, suggesting that selegiline disrupts mitochondrial energy metabolism and contributes to metabolic stress beyond simple MAO-B inhibition. This indicates that selegiline may act as an emerging metabolic checkpoint inhibitor, driving cancer cells into an energy-depleted state that promotes programmed cell death. At present, however, the concept of selegiline as a metabolic checkpoint inhibitor is largely conceptual and supported mainly by preclinical studies; clinical evidence for this role is still limited. Additionally, combinational approaches have provided compelling evidence for selegiline’s therapeutic relevance. In combination with chemotherapy agents, selegiline has shown promise in reducing cancer cell viability (Gross et al., 2020).

Preclinical combination studies provide further support for selegiline’s therapeutic potential. In several models, selegiline enhances the efficacy of standard anticancer agents, including cabazitaxel, abiraterone, and enzalutamide, by sensitizing resistant tumor cells and reducing viability (Antonucci et al., 2021). These findings suggest that selegiline may modulate redox balance and mitochondrial metabolism, particularly in aggressive or treatment-refractory tumors. Importantly, these observations are currently limited to experimental systems and require validation *in vivo.*

Mechanistic insights from cancer models are complemented by observations from neuroprotective studies, where selegiline modulates apoptotic regulators such as Bax and Bcl-2 and preserves mitochondrial integrity (Tábi et al., 2019). While these findings were derived from neuronal systems, they provide indirect support for a conserved role of selegiline in regulating mitochondrial apoptosis, which may also be relevant in cancer cells (Ferretti et al., 2023; Zhao et al., 2013; Naoi et al., 2020). However, direct extrapolation from neuroprotection to oncology should be made cautiously. In breast cancer models, including estrogen receptor-positive and triple-negative subtypes, selegiline induces apoptosis through mechanisms independent of reactive oxygen species, involving inhibition of protein kinase C phosphorylation (Sharif Siam et al., 2020). These data reinforce the existence of MAO-B-independent anticancer pathways and support the potential utility of selegiline in biomarker-defined tumor subsets.

At present, direct clinical evidence for the effects of selegiline in oncology remains limited. No dedicated phase I/II trials of selegiline in cancer patients have been reported. However, clinical studies of other MAO inhibitors provide indirect translational support. In a phase II trial of patients with biochemical recurrent castration-sensitive prostate cancer, the non-selective MAO inhibitor phenelzine produced meaningful declines in prostate-specific antigen (PSA) in a subset of patients, demonstrating that MAO targeting can yield clinical benefit in a specific type of cancer (Gross et al., 2020). While these results cannot be directly extrapolated to selegiline, they support the broader therapeutic rationale for MAO inhibition.

From a translational perspective, these preclinical findings, together with early clinical reports with MAO inhibitors, support selegiline as a plausible metabolic target in various types of cancer. Collectively, these preclinical and emerging clinical findings position selegiline as a promising therapeutic agent in oncology. It appears to reprogram cancer cell metabolism by disrupting mitochondrial energy production and redox homeostasis, while enhancing the efficacy of chemotherapy (Gaur et al., 2019; Devlies et al., 2021). However, more *in vivo* studies and well-designed clinical trials are needed to identify the cancer types that may benefit, define the optimal dosing strategies, clarify the mechanism of action, and establish clinical relevance (Pu et al., 2024). In particular, prospective trials incorporating MAO B expression, metabolic phenotyping, and immune readouts as stratification or exploratory endpoints are lacking and will be essential to move from conceptual metabolic checkpoint modulation to clinically actionable strategies.

### 7.1 Combinational metabolic therapies to enhance Selegiline’s anticancer activity

Rational combination strategies may enhance the anticancer potential of selegiline by exploiting tumor-specific metabolic dependencies and limiting adaptive resistance. Given the heterogeneous metabolic phenotypes observed across cancers, combination regimens should be tailored according to tumor bioenergetic profiles rather than applied uniformly (Guo et al., 2024).

In tumors with strong reliance on OXPHOS, combining selegiline with agents that impair mitochondrial respiration, such as complex I inhibitors or clinical candidates like IACS-010759, may amplify energetic stress and compromise tumor survival (Aisu et al., 2024; Cadassou and Jordheim, 2023; Chen D. et al., 2020). However, such combinations represent a high-risk, high-reward strategy, as excessive mitochondrial inhibition may also affect normal tissues. Careful dose optimization, scheduling, and tumor-selective delivery will therefore be essential to maximize therapeutic benefit while minimizing toxicity. In contrast, glycolysis-dominant (“Warburg-like”) tumors may be more amenable to combinations that concurrently suppress glycolytic flux and MAO-associated metabolic adaptation. Pairing selegiline with glycolysis inhibitors, such as 2-deoxy-D-glucose or lactate dehydrogenase inhibitors, could restrict both major ATP-generating pathways and exacerbate metabolic vulnerability (Luo et al., 2023; Ge et al., 2025). Preclinical studies combining glycolytic and mitochondrial inhibitors support the feasibility of dual metabolic blockade in metabolically flexible tumors (Zhang et al., 2025; Elgendy et al., 2019).

Beyond purely metabolic agents, selegiline may also function as a metabolic sensitizer when combined with conventional therapies. Preclinical evidence suggests that selegiline can enhance tumor responsiveness to chemotherapy and radiotherapy, particularly when administered in a temporally optimized manner (Zhang et al., 2025; Cadassou and Jordheim, 2023; Chen D. et al., 2020). Transient MAO-B inhibition before cytotoxic treatment may increase tumor susceptibility to therapeutic stress while limiting systemic toxicity, although this strategy requires rigorous validation. Finally, combinations incorporating immune-metabolic modulators represent an emerging avenue. Agents such as metformin, which influence cellular energy sensing and immune cell metabolism, may complement selegiline’s metabolic effects and potentially enhance antitumor immunity (Zhu et al., 2024; Gu et al., 2025; Chen et al., 2021). However, given the context-dependent effects of metformin on mitochondrial function, such combinations should be evaluated in models that accurately reflect tumor metabolic state and immune composition. Collectively, these combination strategies underscore the need for biomarker-guided selection based on metabolic phenotype, MAO-B expression, and therapeutic context. While largely supported by preclinical evidence, they provide a rational framework for future translational studies exploring selegiline-based combination regimens in cancer.

## 8 Selegiline as a modulator of immune signaling and the tumor microenvironment

Beyond its tumor-intrinsic metabolic effects, selegiline has emerged as a modulator of immune and stromal components within the TME. By inhibiting MAO-B-associated redox and amine metabolic signaling (as discussed in Section 4), selegiline indirectly -modulates inflammatory tone, immune cell function, and stromal tumor interactions, thereby contributing to a more permissive environment for antitumor immunity (Bakhshalizadeh et al., 2011; Florescu et al., 2023; Tian et al., 2023).

Preclinical studies demonstrate that MAO inhibition attenuates pro-inflammatory cytokine production, including IL-6, TNF-α, and IL-1β, and alters macrophage-associated inflammatory markers, consistent with remodeling of the TME (Tian et al., 2023; Ahmed and Chetia, 2024; Cui et al., 2020). Notably, much of the mechanistic evidence linking monoamine oxidase activity to macrophage polarization derives from MAO-A-focused studies, where MAO-A expression promotes an immunosuppressive M2-like tumor-associated macrophage (TAM) phenotype (Han et al., 2023; Shi et al., 2024). Pharmacological inhibition of MAO activity shifts TAMs toward a more pro-inflammatory, M1-like state and enhances antitumor immune responses. While selegiline selectively targets MAO-B, these findings provide important contextual support for a broader role of MAO-mediated amine metabolism in immune regulation. Recent reviews of MAO B in cancer further highlight that MAO B-driven oxidative stress and amine metabolism can shape an immunosuppressive microenvironment, integrate metabolic and immune regulation, and influence responses to immunotherapies and metabolic drugs (Han et al., 2023).

In addition to its neuropharmacological effects, selegiline, an inhibitor of MAO-B, has demonstrated significant potential to modulate the TME and enhance anti-tumor immunity (Abadi et al., 2021). Selegiline and other nanoformulations may influence the metabolizing process of several biogenic amines by blocking MAO-B. This may affect signalling pathways essential for immune cell activation and function in the TME (Girigoswami and Girigoswami, 2023). MAO-B acts as a specialized immunological checkpoint that controls T cell exhaustion, and inhibition of MAO has recently been shown to improve blocking checkpoint responses (Wang Y.-C. et al., 2021). The findings indicate that selegiline is a potential metabolic-immune modulator that can alter the TME and enhance anti-tumor immunity, in addition to being a neuromodulatory medication (Müller et al., 1998).

In addition to immune cells, stromal components such as CAFs are highly sensitive to metabolic and redox cues within the TME. Inhibition of MAO-B activity may suppress CAF activation, reduce extracellular matrix deposition, and normalize metabolic competition for oxygen and nutrients, thereby alleviating physical and metabolic barriers to immune cell infiltration (Mao et al., 2021; Zhu et al., 2022; Angioni et al., 2021; Zhao et al., 2023). Reduced metabolic stress within the TME can restore cytotoxic T-cell and natural killer (NK) cell function, which are otherwise impaired by high lactate levels, glucose depletion, and oxidative stress (Li M. et al., 2023; Kundu et al., 2024; Yang et al., 2025; Piwocka et al., 2024).

## 9 Challenges and future perspectives of selegiline in cancer therapy

Selegiline shows promising anticancer activity in several preclinical studies, but its clinical use remains challenging (Joung et al., 2023). One major issue is the dose-dependent loss of MAO-B selectivity. At higher doses, selegiline also inhibits MAO-A, which can cause side effects like hypertensive crisis and serotonin toxicity. PET studies in humans confirmed MAO-A inhibition at high concentrations, suggesting that dose adjustment and safety monitoring are important when using selegiline in cancer therapy (Fowler et al., 2015). The limited bioavailability and poor tumor targeting of selegiline reduce its therapeutic efficiency. Oral administration undergoes first-pass metabolism, resulting in variable plasma levels. To address this, researchers are developing nanoparticle- and liposome-based delivery systems to enhance tumor accumulation and reduce systemic toxicity. These systems may allow lower doses while maintaining anticancer activity (Steib et al., 2025; Yang et al., 2023). Another major challenge is the lack of patient selection markers, where tumors show variable expression of MAO-B, and not all cancer types may respond equally to selegiline. Importantly, emerging evidence indicates that both MAO-B-dependent and MAO-B-independent mechanisms contribute to selegiline’s anticancer activity, suggesting that MAO-B expression alone may be insufficient to predict therapeutic response.

Studies in prostate and breast cancer cells have shown that selegiline reduces the expression of GLUT1 and FOXA1, indicating suppression of glycolysis and tumor proliferation (Yang et al., 2020). Identifying tumors with high MAO-B activity or metabolic reprogramming signatures could help select patients who may benefit from this drug. Selegiline also influences key metabolic pathways, including AMPK activation and mitochondrial function (Du et al., 2024). Evidence from metabolic studies shows that it alters energy utilization and reduces oxidative stress. These effects suggest potential combination therapy with drugs targeting glycolysis, fatty acid oxidation, or oxidative phosphorylation (Goenka et al., 2023). However, further studies are needed to understand how cancer cells adapt to these metabolic changes and to identify the best therapeutic combinations. Future research should focus on multi-omics studies to analyze changes in gene, protein, and metabolite levels following selegiline treatment. This approach will help to clarify its molecular targets and resistance mechanisms (Liapopoulos et al., 2025).

Preclinical animal models that simulate tumor microenvironment conditions are needed to evaluate their long-term effects on tumor growth, angiogenesis, and immune modulation (Oriuchi et al., 2020). Designing phase I/II clinical trials with metabolic and safety endpoints, such as lactate levels, Fluorodeoxyglucose-Positron Emission Tomography (FDG-PET) imaging, and MAO activity, will be crucial for translating preclinical findings into clinical benefits (Hirata and Tamaki, 2021). Overall, selegiline has strong potential as a metabolic modulator in cancer therapy. Addressing its pharmacokinetic limitations, improving tumor delivery, and integrating biomarker-based patient selection will determine its success as a repurposed anticancer drug.

### 9.1 Biomarker-guided patient selection for selegiline-based metabolic therapy

Effective patient selection is crucial to the success of metabolic therapies such as selegiline. Candidate predictive biomarkers include tissue MAO-B expression (assessed by immunohistochemistry or RNA profiling), which should be considered an enrichment marker rather than a sole predictor of response, along with metabolic gene signatures (e.g., glycolysis vs. OXPHOS scores), and metabolite levels such as baseline lactate, ATP, and NAD^+^/NADH ratios. Imaging biomarkers offer non-invasive options that can quantify tumor glycolytic activity, helping to identify tumors less reliant on mitochondrial respiration, while novel PET tracers targeting mitochondrial function or redox state (including MAO-B-specific tracers) may pinpoint OXPHOS-dependent or highly oxidized tumors more likely to respond to MAO-B inhibition (Jasper et al., 2023; Villemagne et al., 2022). Integrating these biomarkers into early-phase clinical trials, such as using FDG-PET and metabolic blood markers before and after short-term selegiline treatment, facilitates rapid decisions on whether to advance patient cohort expansion. In addition to predictive markers, pharmacodynamic biomarkers should be monitored, including the changes in tumor lactate or ATP via MR spectroscopy or liquid biopsy, serial FDG-PET or redox-sensitive PET scans to track early metabolic response, and immune cell functional assays (e.g., T-cell proliferation, cytokine profiling) if combined with immunotherapy (Wu et al., 2024). Composite biomarker strategies combining tissue MAO-B, metabolic imaging, and circulating metabolites are likely to be more robust than single markers and should be prospectively validated in phase I/II trials to enable biomarker-driven patient enrichment (Giddabasappa et al., 2025; Grambow-Velilla et al., 2023).

## 10 Conclusion

Selegiline has emerged as a promising metabolic modulator with the potential to influence key pathways that support tumor growth, including redox imbalance, mitochondrial activity, glycolytic dependence, and hypoxia-driven signaling. By selectively inhibiting MAO-B, selegiline reduces hydrogen peroxide-mediated oxidative stress, alters energy utilization, and disrupts the metabolic networks that cancer cells use for survival and resistance to therapy. Its effects extend beyond tumor-intrinsic metabolism and include remodelling of the TME through suppression of inflammatory cytokine production and modulation of macrophage polarization, thereby enhancing antitumor immunity. Preclinical findings also show that selegiline can act through MAO-independent mechanisms by affecting mitochondrial metabolism, ATP production, and apoptotic regulators, indicating a broader metabolic impact than previously recognized. These properties support its potential use in combination with chemotherapy, radiotherapy, metabolic inhibitors, and immunotherapies. However, variable MAO-B expression across cancers, dose-dependent MAO-A inhibition, and pharmacokinetic limitations highlight the need for optimized delivery systems and biomarker-guided patient selection. Overall, selegiline represents a compelling candidate for drug repurposing in oncology, especially in tumors with high oxidative stress and metabolic dysregulation. Advancing multi-omics analyses, targeted delivery technologies, and early-phase clinical trials will be essential to define its therapeutic value as an emerging metabolic checkpoint inhibitor and to translate its preclinical promise into effective cancer therapy.
